# [Corrigendum] Differential regulation of the biosynthesis of glucose transporters by the PI3-K and MAPK pathways of insulin signaling by treatment with novel compounds from *Liriope platyphylla*

**DOI:** 10.3892/ijmm.2026.5840

**Published:** 2026-04-28

**Authors:** Yoen Kyung Lee, Ji Eun Kim, So Hee Nam, Jun Seo Goo, Sun Il Choi, Young Hwan Choi, Chang Jun Bae, Jong Min Woo, Jung Sik Cho, Dae Youn Hwang

Int J Mol Med 27: 319-327, 2011; DOI: 10.3892/ijmm.2010.581

Following the publication of the above article, an interested reader drew to the authors' attention that the β-actin blots featured for the western blots in Figs. 5A and 8A were apparently the same, even though the samples came from different tissues (liver and brain, respectively). Upon re-examining their original data, the authors have realized that the control blots were erroneously selected for Fig. 8A; moreover, the same processing error was made with the control blots for Fig. 6A (duplicated from those for Fig. 4A) and Fig. 9A (duplicated from Fig. 7A).

The revised versions of Figs. 6, 8 and 9, now showing the correct control western blots in Figs. 6A, 8A and 9A, are shown opposite and on the subsequent page. Note that the errors made in assembling these figures did not affect the overall conclusions reported in the paper. All the authors agree with the publication of this corrigendum, and are grateful to the Editor of *International Journal of Molecular Medicine* for granting them the opportunity to publish this. Furthermore, they apologize to the readership for any inconvenience caused.

## Figures and Tables

**Figure 6:**
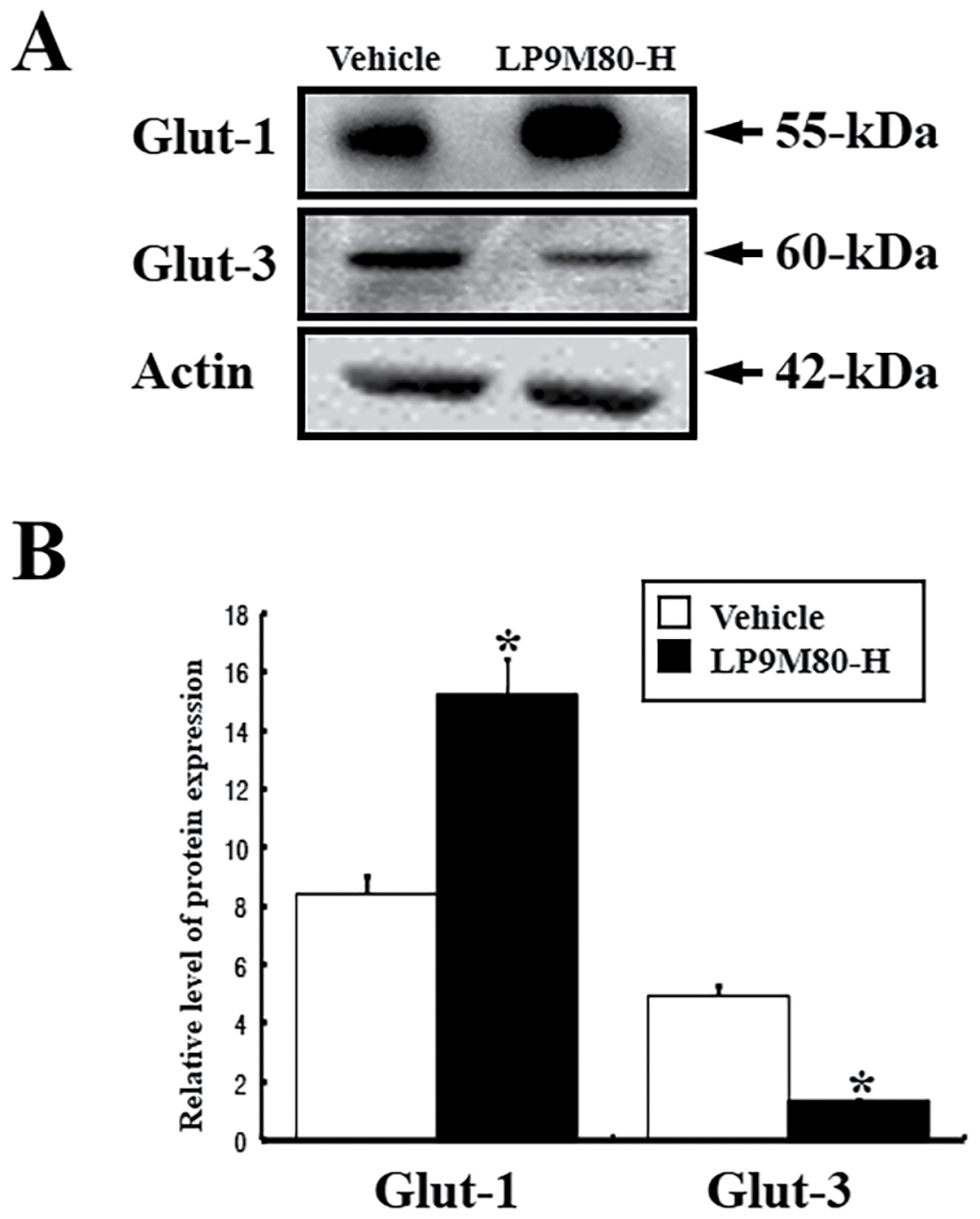
Expression levels of Glut-1 and Glut-3 in the livers of vehicle-treated and LP9M80-H-treated mice. The Glut-1 and Glut-3 protein expression in the liver was detected with anti-Glut-1, anti-Glut-3 primary antibodies, and horseradish peroxidase-conjugated goat anti-rabbit IgG, as described in Materials and methods. The intensity of the Glut protein was calculated using an imazing densitometer. The values are the mean ± SD. ^*^p<0.05 is the significance level compared to the vehicle-treated group.

**Figure 8:**
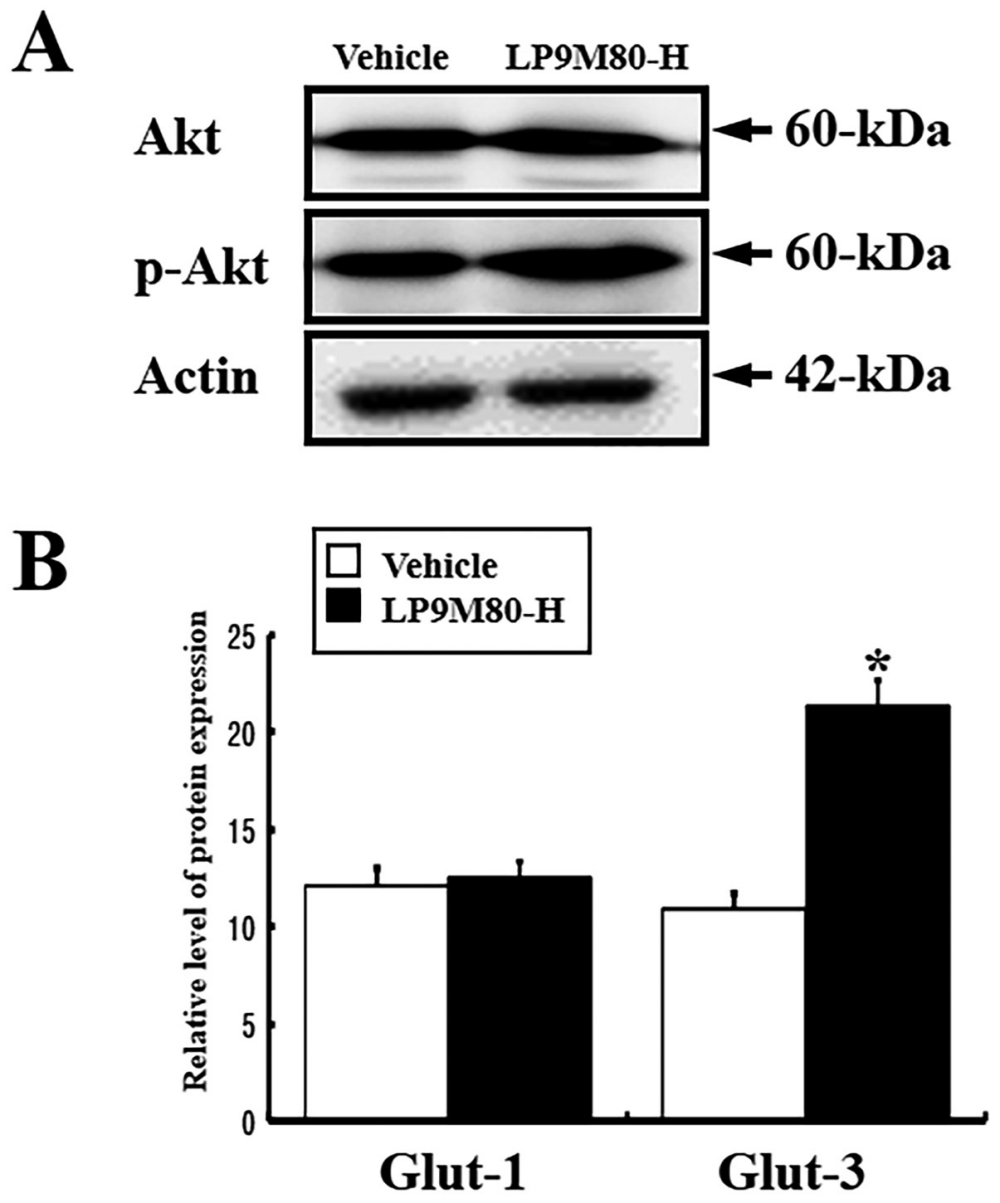
Phosphorylation levels of Akt in the brains of vehicle-treated and LP9M80-H-treated mice. Cell lysates were prepared from brain tissues of vehicle-treated and LP9M80-H-treated mice, as described in Materials and methods. Protein, 50 μg per sample, was immunoblotted with antibodies for each protein. Three samples were assayed in triplicate using Western blotting. The values are the mean ± SD. ^*^p<0.05 is the significance level compared to the vehicle-treated group.

**Figure 9:**
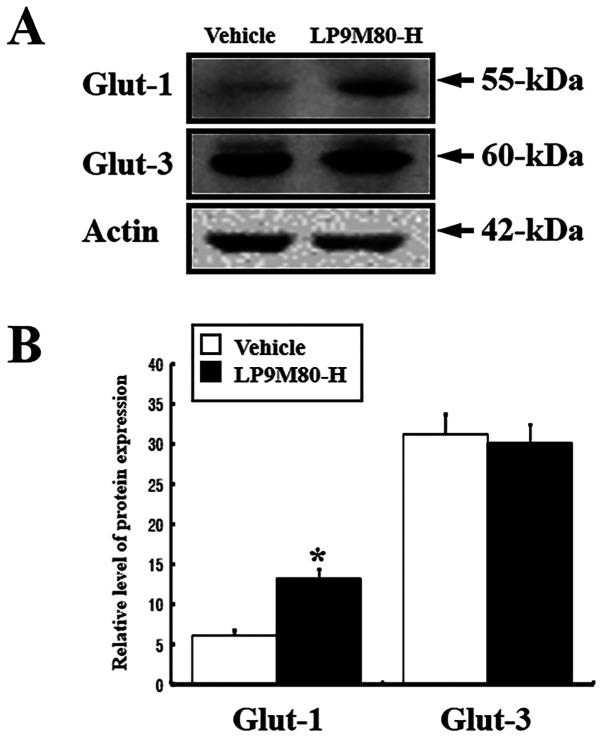
Expression levels of Glut-1 and Glut-3 in the brain of vehicle-treated and LP9M80-H-treated mice. The Glut-1 and Glut-3 protein expression in the brain was detected with anti-Glut-1, anti-Glut-3 primary antibodies, and horseradish peroxidase-conjugated goat anti-rabbit IgG as described in Materials and methods. The intensity of the Glut protein was calculated using an imaging densitometer. The values are the mean ± SD. ^*^p<0.05 is the significance level compared to the vehicle-treated group.

